# Fano-like resonance emerging from magnetic and electric plasmon mode coupling in small arrays of gold particles

**DOI:** 10.1038/srep32061

**Published:** 2016-09-01

**Authors:** Saïd Bakhti, Alexandre V. Tishchenko, Xavier Zambrana-Puyalto, Nicolas Bonod, Scott D. Dhuey, P. James Schuck, Stefano Cabrini, Selim Alayoglu, Nathalie Destouches

**Affiliations:** 1 Univ Lyon, UJM‐Saint‐Etienne, CNRS, Institut d Optique Graduate School, Laboratoire Hubert Curien UMR 5516, F‐42023, SAINT‐ETIENNE, France; 2 Aix Marseille Univ, CNRS, Centrale Marseille, Institut Fresnel, Marseille, France; 3 Molecular Foundry, Lawrence Berkeley National Lab, Berkeley, CA, USA; 4 Chemical Sciences Division, Lawrence Berkeley National Lab, Berkeley, CA, USA

## Abstract

In this work we theoretically and experimentally analyze the resonant behavior of individual 3 × 3 gold particle oligomers illuminated under normal and oblique incidence. While this structure hosts both dipolar and quadrupolar electric and magnetic delocalized modes, only dipolar electric and quadrupolar magnetic modes remain at normal incidence. These modes couple into a strongly asymmetric spectral response typical of a Fano-like resonance. In the basis of the coupled mode theory, an analytical representation of the optical extinction in terms of singular functions is used to identify the hybrid modes emerging from the electric and magnetic mode coupling and to interpret the asymmetric line profiles. Especially, we demonstrate that the characteristic Fano line shape results from the spectral interference of a broad hybrid mode with a sharp one. This structure presents a special feature in which the electric field intensity is confined on different lines of the oligomer depending on the illumination wavelength relative to the Fano dip. This Fano-type resonance is experimentally observed performing extinction cross section measurements on arrays of gold nano-disks. The vanishing of the Fano dip when increasing the incidence angle is also experimentally observed in accordance with numerical simulations.

The electromagnetic excitation of conduction electrons in metallic nanoparticles results in the well-known localized surface plasmon resonances (LSPRs)^1^,^2^,^3^. Acting as optical resonators^4^, metallic nanoparticles exhibit highly tunable optical properties through a control of their geometry, spatial organization and environment^5^,^6^. Notably, the sensitivity provided by the coupling effects and the underlying hybridization of resonant modes^7^,^8^,^9^ presents a great potential for light manipulation at sub-wavelength scales. The control of near- and far-field behavior and the high spectral sensitivity of LSPRs to the local environment make such materials particularly suitable for a broad range of applications^10^,^11^,^12^,^13^.

Interestingly, particular arrangements of metal nanoparticles give rise to magnetic plasmon modes^14^. This term refers to collective plasmon modes that generate a circulating displacement current inducing a magnetic behavior in the structure^15^. As an example, ring type planar arrangements of metal nanoparticles can support magnetic modes characterized by the formation of a dipolar magnetic moment orthogonal to the structure^16^,^17^. The coexistence of both magnetic and electric resonance modes in nanometer sized structures is the keystone for different interesting phenomena such as dual particles^18^, chirality enhancement^19^ or negative index metamaterials^16^,^20^,^21^,^22^.

In some cases, coupling effects in plasmonic structures may lead to the formation of Fano-type resonances^23^, resonances that exhibit asymmetric spectral line profiles. The emergence of these atypical line shapes in plasmonic structures has been theoretically investigated and interpreted through coupled mechanical oscillator models^24^,^25^,^26^, the coupled mode theory^27^,^28^,^29^,^30^ or an eigenvalue analysis derived from quasi-static^31^ or coupled-dipole^32^ approximation. Fano resonances are generally attributed to an interplay between a bright and a dark mode of the system^25^,^33^. These two subclasses of hybrid modes are classified according to their ability to couple with plane waves. Typically, the bright modes have significant dipolar character, highly overlap with the incident radiation and are highly radiative. In contrast, the dark modes are characterized by a vanishing overlap with the incident excitation, making them non-radiative and appearing as purely near-field or trapped modes^34^. Several works demonstrated that Fano-type resonances result from the interaction between a superradiant (highly radiative) and a subradiant (poorly radiative) mode^31^,^32^,^35^,^36^,^37^,^38^. The design of Fano resonances involving both electric and magnetic type modes appears as an effective way to enhance the magnetic response of plasmonic structures^39^. In this way, a Fano resonance obtained via the interplay of dipolar electric and magnetic modes has been recently achieved on a gold nano-ring when geometrical asymmetries are induced in the structure^40^.

In this work we theoretically characterize a planar oligomer of gold nanoparticles, consisting of a regular array of 3 × 3 particles that presents a Fano-like resonance when illuminated under normal incidence. In the basis of the fully analytical Mie theory generalized to interacting particles, the electromagnetic field scattered by the overall structure is expanded in terms of a multipolar basis. As a result, the oligomer is considered as an effective scattering particle. Resonances in the different multipolar contributions to the total extinction are then considered in order to investigate the origin of the Fano-like resonance. We rely on the coupled mode theory to interpret the Fano-type resonance as resulting from a hybridization process between electric and magnetic resonant modes of the structure. The effect of the structure size as well as the illumination on the oligomer optical response is then explored. The near-field effects, especially magnetic and electric field enhancements on the structure, at normal incidence are also theoretically studied and interpreted according to the previous analysis. Experimental characterizations of gold nano-disk arrays will also be carried out to confirm the effect of the incident angle on the emergence of a Fano-like resonance. To the best of our knowledge, Fano-type resonances on a single plasmonic oligomer under normal incidence have never been reported from the coupling between an electric and a magnetic mode. This result could lead to a revival of interest for this kind of structure, particularly as building blocks for optical metamaterials.

## Results and Discussion

We theoretically investigate the optical properties of a finite array of gold nanoparticles. All results are obtained using the Mie theory generalized to interacting particles^41^,^42^, allowing for a rigorous computation of the total near and far fields scattered by the structure. This model also permits to determine the contribution of electric and magnetic multipoles to the total optical cross-sections of the system (see Methods).

**Figure 1:**
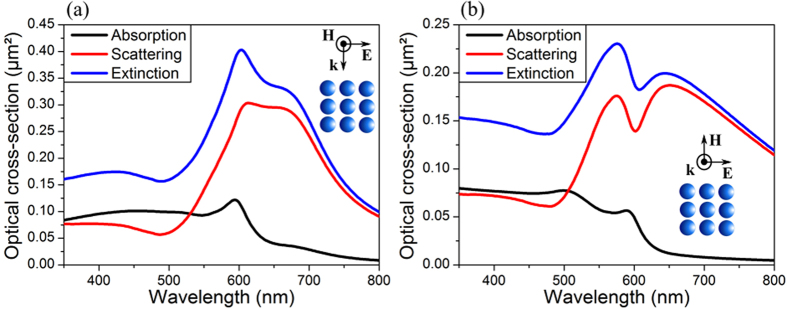
Optical cross-sections (calculated using Mie theory) of a periodic array of 3 × 3 gold nanoparticles of 50 nm in radius in vacuum. The gap between adjacent particles is fixed to 10 nm and the s-polarized incident plane wave is propagating at (**a**) grazing or (**b**) normal incidence.

Let us consider a 3 × 3 periodic array of gold nano-spheres in vacuum, 50 nm in radius, with a gap of 10 nm between the particles. As shown in Fig. 1, the optical cross-sections of the structure strongly depend on the direction of propagation of an s-polarized incident plane wave. A propagating direction parallel to the structure plane induces at least two resonance peaks in the optical spectra (Fig. 1a). For a normal incidence (Fig. 1b), the optical cross-sections exhibit asymmetric line profiles characteristic of a Fano-like resonance. The characteristic dip in the scattering and extinction spectra is centered at about 600 nm, where a resonance peak appears in the absorption spectrum.

By expanding the total field scattered by the whole structure in a multipolar basis (see Methods), we access the oligomer’s scattering coefficients *f*_*mn*_ and *g*_*mn*_ related to magnetic and electric multipolar contributions to the total radiation respectively. With such an expansion, it is possible to consider the whole structure as a unique scattering object virtually bounded by the smallest sphere enclosing the structure. Here, we use the term multipole to refer to the different n orders of the expansion, e.g. n = 1 dipolar, n = 2 quadrupolar, etc. The scattering coefficients are computed in order to identify the nature of the different optical resonances sustained by the structure and particularly those at the origin of the Fano resonance. In the following, we consider the contribution of these different multipoles to the total extinction.

**Figure 2:**
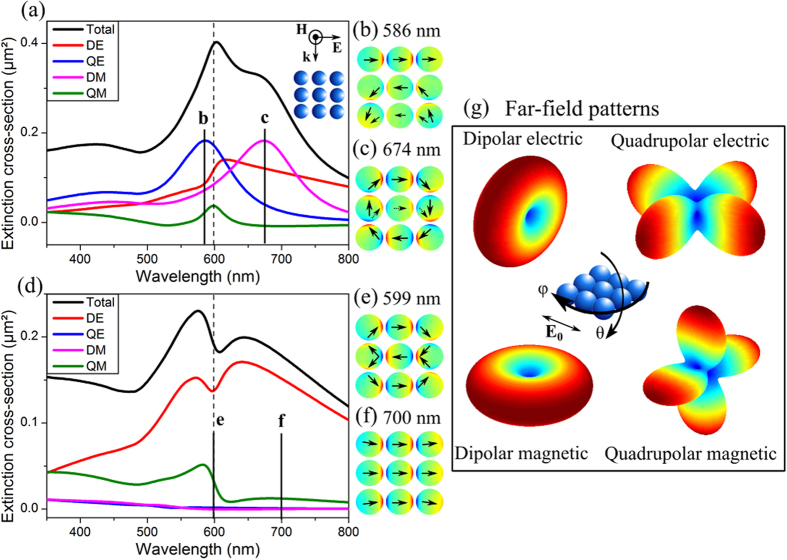
Contribution of different magnetic and electric multipoles to the total extinction in the case of (**a**) grazing and (**d**) normal incidence. (**b**,**c**,**e**,**f**) are surface charge density maps on the particle surface plotted at different wavelengths. (**g**) displays the electric far-field intensity pattern of magnetic and electric modes. All the spectra, surface charge mapping and far-field patterns have been calculated analytically.

For a grazing s-polarized plane wave (Fig. 2a), it appears that both dipolar and quadrupolar electric and magnetic multipoles have a resonant behavior and mainly contribute to the total extinction. More precisely, it is found from the calculation of the different scattering coefficients that only a couple of m orders are dominant: *m* = 0 for the dipolar magnetic (DM), *m* = ±1 for the quadrupolar magnetic (QM), *m* = ±1 for the dipolar electric (DE) and *m* = ±2 for the quadrupolar electric (QE) multipole (see Fig. S1 in Supplementary Information). In the cases where two m orders are mainly involved, it is also found that their corresponding scattering coefficients are either equal or opposite. This indicates that for a given n order, the dominant m orders present resonances at same frequencies. The total extinction cross-section does not exhibit resonances with clear asymmetric line profiles (see Fig. 2a). However, QE, DM and QM contributions exhibit symmetric resonances whereas the DE multipole clearly sustains a non-Lorentzian resonance line shape. Surface charge density distributions on the particles and the deduced electric moments provide additional information on the different resonances in terms of dipolar orientations of the particles. The surface charge densities are obtained by computing the discontinuity of the radial electric field on the surface of the particle. As an example, calculations are done at wavelengths where the resonance peaks in the QE and DM multipoles are maximal (Fig. 2b,c). At 586 nm, although the QE multipole is predominant, the contributions of several other multipoles are non-negligible and the map shows a complex surface charge density distribution. At 674 nm the resonance in the DM multipole clearly predominates over the DE contribution and an electric current loop along the external particles of the structure emerges from the surface charge density map (Fig. 2c).

In the case of normal incidence (Fig. 2d), the QE and DM contributions vanish and only the DE and QM multipoles contribute to the total extinction. The computation of the scattering coefficients (Fig. S2 in Supplementary information) shows that the same m orders are contributing to the multipoles. The surface charge density map obtained at the dip position, 599 nm (Fig. 2e), shows a double electric current loop in the structure that brings out the quadrupolar magnetic contribution. At longer wavelengths (700 nm) where the DE multipole predominates, a dipolar pattern appears in the surface charge density map (Fig. 2f). Each electric or magnetic multipole of the structure exhibits specific spectral far-field features, which can be illustrated by computing the angular distribution of the scattered intensity (Fig. 2g). Whatever the multipole considered, the shape of the scattered intensity pattern is independent of the excitation wavelength and the incidence angle. Only the amplitude and phase of the multipole far field vary with the wavelength and the incidence angle, giving rise to various scattered intensity patterns when summing all contributions. Thereby, under normal incidence, light scattering is maximal in the direction perpendicular to the incident wave vector and polarization (see Fig. S4 in Supplementary information). It predominates in the backward or forward direction depending on the wavelength when illuminating the structure under grazing incidence (see Fig. S4 in Supplementary Information).

Now in order to interpret the resonance behavior of the structure and to elucidate the origin of the Fano-like resonance, we use the coupled mode theory^43^ (CMT) to provide a phenomenological approach on the different coupling paths between the incident light and the resonance modes of the structure. According to the CMT, the optical response of a resonant structure can be represented as a collection of resonant modes subjected to a driven excitation that could possibly experience mutual coupling effects. Then a single spherical particle sustains several resonant modes only coupled to the exciting light and without any interplay between them. In turn, a collection of particles may sustain some so called hybrid modes, resulting from the scattering induced coupling between the plasmon resonances of the different particles^8^,^44^. In the present case we do not treat the structure as a collection of nine mutually coupled particles, which would require a too tedious analysis.

Considering the oligomer as an effective scattering particle, the different multipoles can be viewed as its different radiative channels resulting from a plane wave excitation. In the light of the previous observations, we assume that these multipoles can be assimilated to the delocalized resonance modes of the structure, directly coupled to the incident plane wave and possibly hybridized (i.e. mutually coupled). In this sense, the QE and DM modes (at grazing incidence) are both only coupled to the driving excitation without mutual coupling, since they present a Lorentzian shaped resonance centered at independent wavelengths (Fig. 2a). In turn and especially at normal incidence, the DE and QM modes are spectrally overlapped and have asymmetric line shapes (Fig. 2d), that suggest their strong interplay. Using the CMT, we aim in the following to demonstrate this behavior as resulting from hybridization of the DE and QM modes.

According to the CMT, the temporal amplitudes *a*_1_ and *a*_2_ of two mutually coupled resonance modes upon a driving excitation *f*_0_ can be described using the coupled equations^30^


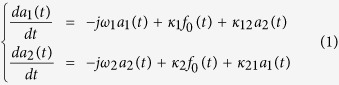


where *κ*_*i*_ is the coupling coefficient of the i^th^ mode with the driving excitation, *ω*_*i*_ its complex valued angular eigenfrequency, *κ*_12_ and *κ*_21_ are the mutual coupling coefficients. It has to be noted here that the real part of a complex valued angular frequency corresponds to the associated resonance position while its imaginary part corresponds to its half-width at half-maximum (HWHM) and is related to the mode losses. Considering the amplitudes in equation (1) oscillating at the angular frequency *ω*, we can rewrite them as *a*_*i*_(*t*) = *ã*_*i*_(*t*)exp(−*jωt*) and 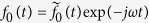, and a steady-state solution of equation (1) can be found with 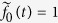 (corresponding to a plane wave excitation):


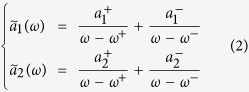


The frequency domain resonance amplitude ã_*i*_(ω) of the i^th^ mode is then formally expressed as a sum of two singular functions of the angular frequency, that are characterized by their complex poles (*ω*^+^ and *ω*^−^) and amplitude constants (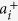 and 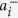). The solutions expressed in equation 2 can be interpreted in terms of hybridization, where the system presents two resonance modes (defined as hybrid modes and characterized by their angular eigenfrequencies *ω*^+^ and *ω*^−^) contributing to the spectral response of the two mutually coupled modes.

In order to demonstrate the hybridization of the DE and QM multipolar contributions to the total extinction of the structure, we apply the CMT to the resonant behavior observed in the complex valued extinction of the electric (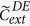) and magnetic (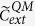) multipoles, whose imaginary part corresponds to their contribution to the total extinction cross-section. The concept of complex extinction (see Methods for more details), recently outlined in ref. 30, is well suited to investigate the hybridization of coupled plasmon resonances.

**Figure 3:**
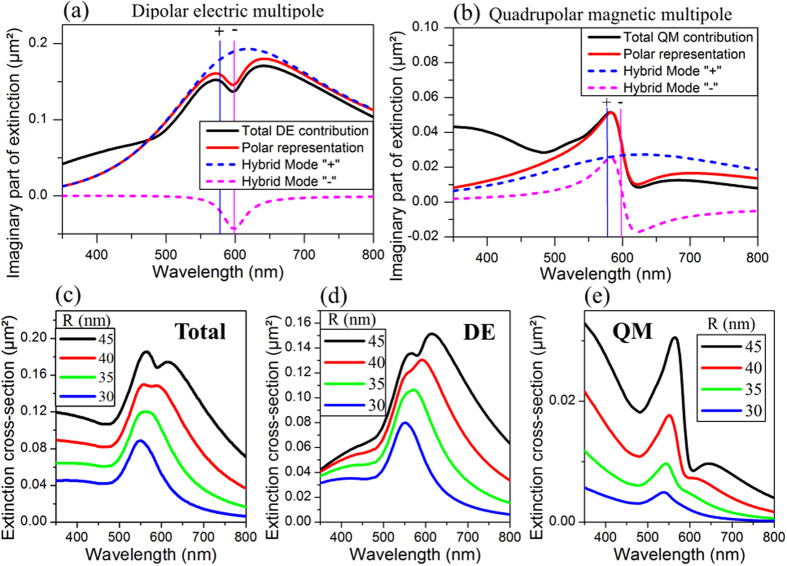
Polar decomposition (with the hybrid mode contributions extracted from analytical Mie calculations) of the (**a**) electric and (**b**) magnetic multipoles’ imaginary part of complex valued extinction, with the hybrid mode resonance positions located by a vertical line. (**c**) Total extinction of the structure with the contribution of the (**d**) dipolar electric and (**e**) quadrupolar magnetic multipoles, for different radiuses of particles (the ratio between the radius and the gap between the particles being fixed constant to 5). (**c–e**) are calculated using Mie theory.

Anticipating the coupling between DE and QM multipolar contributions, their complex extinction should be mainly described by the sum of two singular functions resonant at the same frequencies as described in Eq. (2). A numerical algorithm (detailed in ref. 45) was thus used to extract from the two extinction spectra, computed rigorously by the extended Mie theory, the amplitudes and the angular frequencies of such singular functions. As an evidence of the coupling effect, the complex angular frequencies of the two singular functions extracted from each multipole analysis are exactly the same. We can then identify the two singular functions as being the hybrid modes originating from the coupling phenomenon. These hybrid modes resonate at 577 nm (mode “+”) and 597 nm (mode “−”), with a HWHM much broader for the mode “+” (209 nm) than for the mode “−” (19 nm). We plot in Fig. 3 the contribution of these hybrid modes to the complex extinction’s imaginary part of the DE (Fig. 3a) and QM (Fig. 3b) multipoles. The sum of these contributions fits well with the resonance profiles obtained rigorously in the resonance spectral range. As a consequence, we can conclude that 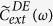 (substituted for 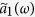) and 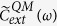 (substituted for *ã*_2_(*ω*)) satisfy equation (2), which validates our initial assumption of hybridization between the two contributions.

Finally, the Fano-like resonance of the structure results from the spectral overlapping of the DE and QM multipoles. If the Fano-like resonance line profile can be fully interpreted in terms of spectral overlapping of resonant modes, the asymmetry of the latter can be described as mostly originating from coupling (hybridization) processes in the structure. In general, the nanoparticle size and spatial arrangement as well as the illumination conditions affect both the direct excitation and the mutual coupling between resonant modes, which in turn fixes the overall optical response of the plasmonic structure. As an example, when scaling down the oligomer’s size (i.e. decreasing the nanoparticle size while keeping the radius to gap ratio equal to 5) under normal incidence, the characteristic Fano dip of the total extinction spectrum gradually vanishes (Fig. 3c) and transforms into a single Lorentzian shaped resonance band. The DE (Fig. 3d) and QM (Fig. 3e) multipolar contributions also show a transition from asymmetric to symmetric line profiles while decreasing the structure size. This result indicates that a minimal structure size is required for the emergence of a Fano-type resonance. The changes in the resonance line profiles while scaling down the oligomer size can be interpreted as a decrease in the mutual coupling strength between the DE and QM multipoles. In a small enough structure (e.g. having 30 nm in radius particles), the coupling between these multipoles is no longer strong enough to present asymmetric line shapes. Then, the DE and QM modes tend to present a Lorentzian line shape characteristic from independent resonators.

**Figure 4:**
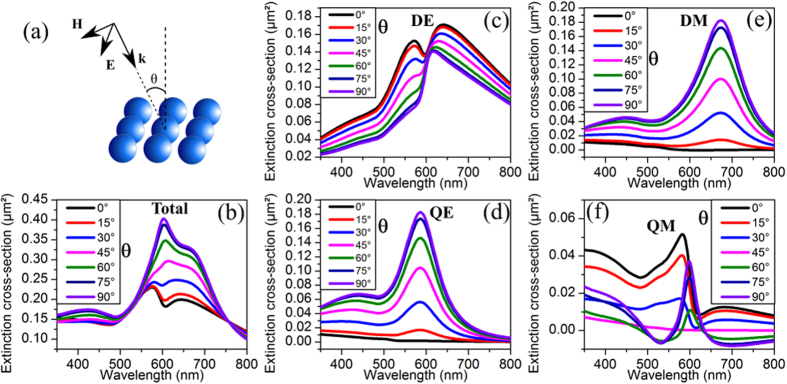
(**a**) Geometry of the structure illumination. (**b**) Total extinction cross-section of the structure versus the incident angle with the contribution to this extinction of (**c**) the dipolar electric, (**d**) the quadrupolar electric, (**e**) the dipolar magnetic and (**f**) the quadrupolar magnetic modes. All spectra are obtained using the analytical Mie theory.

Changing the illumination conditions also significantly affects the structure’s optical response. Considering the initial structure, the Fano dip obtained at normal incidence progressively vanishes from the total extinction (Fig. 4b) when increasing the incidence angle θ (illustrated in Fig. 4a). This evolution of the extinction spectrum appears as mainly being a consequence of changes in the interplay between the incident light and the different multipoles sustained by the structure. So, the QE (Fig. 4d) and DM (Fig. 4e) multipole amplitudes increase with the incident angle, as a consequence of an increase of their coupling strength with the incident plane wave. Concerning the DE (Fig. 4c) and QM (Fig. 4f) multipoles, their spectral line profile significantly changes with the incidence angle. Particularly for the QM mode, its line shape appears to be more symmetric for large incidence angles. This behavior can be qualitatively interpreted as changes in the mutual coupling strength between the QM and DE modes combined with a change in their direct coupling with the incident light.

**Figure 5:**
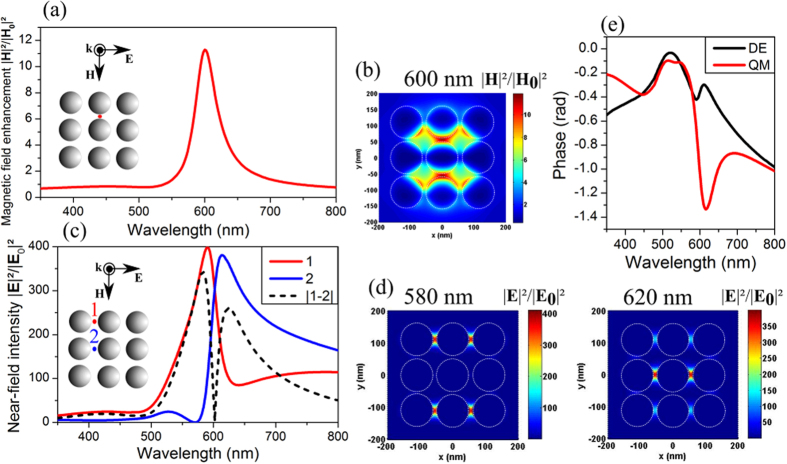
(**a**) Magnetic field enhancement in the structure with (**b**) the magnetic field mapping at 600 nm. (**c**) Near-field intensities and contrast between the two first lines of the array versus wavelength with (**d**) near-field mapping at 580 nm and 620 nm. (**e**) Phase of the complex extinction of dipolar electric and quadrupolar magnetic multipoles. All the near-fields and phases are calculated using the analytical Mie theory.

An interesting feature of such a system is the local magnetic field enhancement that occurs in the spectral range of the Fano dip (around 600 nm) (Fig. 5a). At this resonance position, the magnetic near-field intensity is distributed over the whole structure (Fig. 5b). A rapid change in the distribution of the electric near-field enhancement also takes place when crossing the magnetic resonance position. If we consider lines of particles parallel to the incident polarization, the total near electric field is confined in external lines on the blue side of the Fano dip and in the central line on the red side of the same dip. The spatial confinement of light enhancement can be optimized by computing the spectral variations of the total near-field intensity in both kinds of lines as shown in Fig. 5c where we observe a sharp decrease (increase) in the near-field intensity of the external (central, respectively) line around the resonance position. Then, the contrast between these two near-field intensities (Fig. 5b) appears maximal on either side of the resonance, at 580 and 620 nm. The near-field intensity maps computed at these two wavelengths are shown in Fig. 5d and confirm the efficient confinement and enhancement of light in different lines of the structure.

The tunability of hot-spot localization with the wavelength has been pointed out in the case of metallic nanoparticle chains^46^,^47^,^48^ or small arrays. While an asymmetric longitudinal mode causes near-field localization in a single gap of a trimer^47^, retardation effects induce an efficient confinement of light in both end particles of a longer chain^46^ as well as for small arrays of weakly coupled particles^48^. In our case, light focusing in the central or external lines directly results from the spectral overlapping of the DE and QM resonances with different relative phases and amplitudes. In order to characterize this behavior we use the complex valued extinction of the two multipoles, whose phase corresponds to their resonance phase relative plane wave (see Supplementary Information). The dipolar electric multipole is characterized by an identical electric moment orientation of all the particles in the structure (Fig. 2f). Moreover, the phase of oscillating dipoles for this mode, mainly dominated by the hybrid mode “+”, varies slightly around the Fano dip (Fig. 5e). On the contrary, the magnetic multipole is characterized by opposite resulting electric moments in the central and external lines. The phase of the charge oscillations in this case is mainly imposed by the sharp mode “−”, leading to a fast phase change around the Fano dip (Fig. 5e). The relative phase shift between the two electric and magnetic multipoles consequently has a sharp variation from one side of the Fano dip to the other. On the blue side, the dipolar moments of both modes are in phase on the external lines and out-of-phase on the central line resulting in a total field enhancement on the external lines. On the red side the situation is reversed and a better phase matching occurs in the central line.

**Figure 6:**
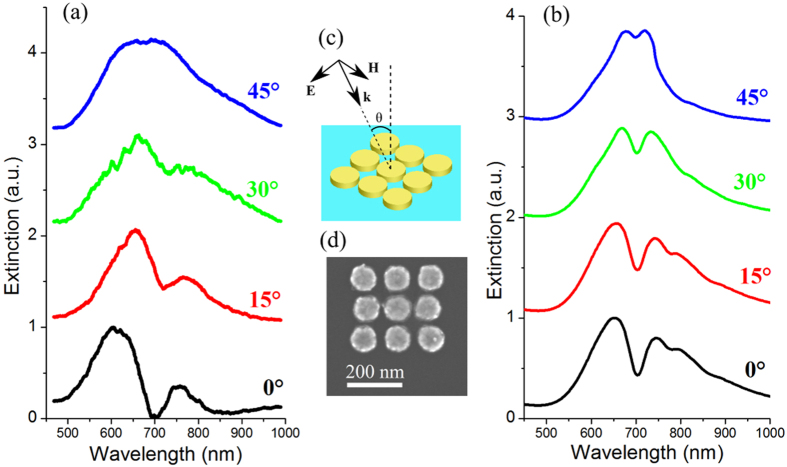
(**a**) Experimental measurement of the optical extinction and (**b**) numerical simulation (using Lumerical FDTD software) of the optical transmission of an array of gold nano-disks. (**c**) Experimental and simulation configuration. (**d**) Scanning electron microscopy micrograph of the structure.

The spectral behavior of such plasmonic oligomers has been experimentally characterized using arrays of gold cylinders. These structures, fabricated using electron beam lithography, consist of a series of 3 × 3 arrays of gold/chromium bi-metallic disks (the chromium acting as an adhesive layer). The disks have a diameter of 100 nm and a height of 5 nm (chromium) + 25 nm (gold) and have been deposited on a quartz substrate. The particular structure considered here is slightly different from the theoretical case which has been previously thoroughly studied. The differences concern the presence of a plane interface (the substrate), the geometry of particles (cylinders rather than spheres) and finally changes in the gaps between particles. For the external lines, the gap between particles is 20 nm, whereas it is 10 nm in the central line (Fig. 6d). Compared to a regular array, this configuration reduces the number of gold bridges remaining between particles after the lithography process (due to the small gap/diameter ratio) and then ensures the excitation of proper optical resonances.

The spectral behavior of samples shown in Fig. 6d has been measured on single oligomers at various incidence angles using a s-polarized plane wave (Fig. 6c) with the set-up described in the Method section. At normal incidence (Fig. 6a), the extinction spectrum exhibits a Fano dip at 700 nm, this dip being attenuated by increasing the incidence angle and vanishes at 45°. This behavior is similar to the one theoretically observed in an array of spheres (Fig. 4b). These measurements are supported by Finite Difference Time Domain (FDTD) simulations of the structure’s transmission spectra (Fig. 6b, performed with Lumerical software) that take into account the particles geometry (with gold and chromium disks) and the substrate. A good agreement with the Fano dip position can be observed between experimental results and simulations, as well as its attenuation with the incidence angle. Numerical simulations also show a similar behavior around the Fano dip of both the electric and magnetic near fields in the experimental structure (shown in Fig. S5 in Supplementary Information), compared to a regular array of spheres in air. This indicates that the interplay between electric and magnetic modes in this structure is also at the origin of the Fano-type resonance. We provide in Supplementary Information details on changes in the structure resonance behavior from the theoretically studied oligomer to the experimental one.

**Figure 7:**
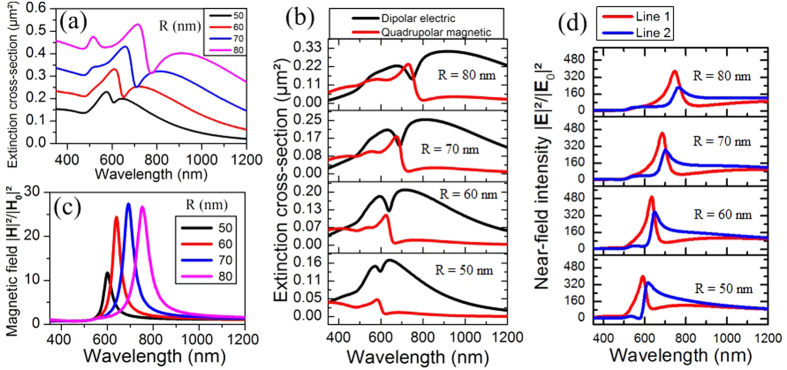
(**a**) Total extinction cross-section of the structure for various particle sizes and keeping the ratio of radius on gap constant and equal to 5. (**b**) Contribution of the dipolar electric and the quadrupolar magnetic modes to the total extinction, for various particle sizes. (**c**) Magnetic field enhancement in the structure and (**d**) near-field intensity on the two first lines for different particle sizes. All these spectra are obtained using the analytical Mie theory.

Having shown previously that a minimal oligomer size is required for the emergence of a Fano-like resonance, we explore here how the structure optical properties evolve when scaling up its dimensions. When keeping the radius to gap ratio constant and equal to 5, the increase in the particle size results in a redshift and a broadening of the Fano dip in the total extinction spectra (Fig. 7a). The contribution of the different multipoles to the total extinction (Fig. 7b) shows a decrease in the relative amplitude of the dipolar electric mode compared to the quadrupolar magnetic one. This indicates an increase in the mutual coupling strength between these two modes. Paying attention to the near-fields, the magnetic field enhancement (Fig. 7c) appears as being optimal for the structure having 70 nm in radius particles. In turn, the near-field intensity appears as optimal, for both its amplitude and contrast between the different lines on both sides of the Fano dip, for the oligomer having 60 nm in radius. This behavior highlights the importance of the oligomer size on its optical performances, which is a crucial element in the structure design considering further particular applications.

Interestingly, the interplay between electric and magnetic modes also explains the formation of Fano-like resonances in other plasmonic oligomers. As an example, the widely studied heptamers^49^ exhibit under normal incidence the same resonance behavior of their dipolar electric and quadrupolar magnetic multipolar contributions (see Fig. S8 in Supplementary information). The bright and dark modes previously identified in the literature^49^,^50^ can thereby be related to the dipolar electric and the quadrupolar magnetic modes of the structure, respectively. This explains especially the dipolar moment orientations obtained for the dark mode, which reproduces a dual electric current loop generating a quadrupolar magnetic moment.

## Conclusion

We characterized the Fano-like resonance of a 3 × 3 array of gold nanoparticles illuminated at normal incidence, as resulting from the coupling between the dipolar electric and quadrupolar magnetic multipolar contributions to the total extinction of the structure.

On the basis of the coupled mode theory, the asymmetric Fano line shape was analyzed by using an analytical representation of the structure and by expressing the complex optical extinction as a sum of singular functions of the angular frequency. This analysis brings a clear evidence of hybridization of the electric and magnetic multipolar contributions. The unusual phase behavior of the two hybrid modes formed by the coupling process explains the non-Lorentzian resonance profiles and the characteristic Fano dip results from their spectral interferences.

As theoretically highlighted, the structure supports a local enhancement of the magnetic field, but also the special feature to confine the electric near-field intensity in different lines depending on the illumination wavelength relative to the Fano dip under normal incidence. This interesting property was assumed to result from constructive or destructive contributions of electric and magnetic multipoles to the total near-field on the different lines of the array. An experimental characterization of the Fano-type resonance has been performed through the optical extinction measurement of gold nano-disk arrays. In accordance to simulations, the Fano dip vanishes when increasing the incidence angle from the normal.

Based on the fully analytical Mie theory, the methodology presented in this work can be applied to the analysis of the resonance behavior of arbitrary structures composed of nano-spheres. The results obtained here are retrieved in other plasmonic oligomers. We pointed out that the Fano-type resonance observed in heptamers also results from electric and magnetic mode interplay. This could extend the application range of this kind of structures, particularly for the possibility to generate artificial magnetism at optical frequencies.

## Methods

### Modeling details

All simulations are performed using the Mie theory generalized to interacting particles, that allows for solving rigorously the multiple scattering problem^41^,^42^,^51^. A Drude model is used to model the gold refractive index:


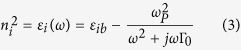


where Γ_0_ is the damping constant of bulk gold (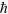
Γ_0_ = 70.9 *meV*), *ω*_*P*_ is the plasma angular frequency of gold (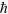
*ω*_*p*_ = 9.02 *eV*). *ε*_*ib*_ is the contribution of interband transitions computed by using Kramers-Kronig relations on the experimentally obtained refractive index^1^. We note that numerical simulations are in turn performed using the experimental values of the refractive index given in ref. 52, and provided in Supplementary Information (Fig. S9) together with the values obtained with the semi-analytical Drude model.

Once the scattering problem is solved, the total electric field scattered by the structure is represented in terms of vector wave function decomposition:


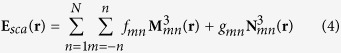


where 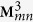 and 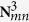 are outgoing spherical wave functions (solutions of the vector wave equation) weighted by the scattering expansion coefficients *f*_*mn*_ and *g*_*mn*_ respectively. The superscript “3” on these wave functions corresponds to the use of spherical Hankel functions of the first kind as radial functions, that ensures the Silver-Müller radiation condition for the scattered field^53^. The optical cross-sections of the structure are expressed in function of these expansion coefficients^54^:


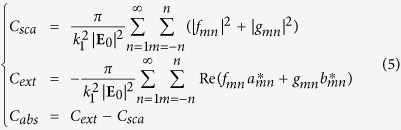


with *k*_1_ the wavenumber in the surrounding medium, **E**_0_ the incident electric field, *a*_*mn*_ and *b*_*mn*_ the incident field expansion coefficients. The representation (4) directly provides the contribution of the multipolar electric and magnetic modes. Then the terms 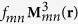 with *n* = 1 correspond to the dipolar magnetic (DM) contribution to the total scattering, with *n* = 2 to the quadrupolar magnetic (QM) contribution, and so on. Moreover, the terms 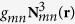 with *n* = 1 correspond to the dipolar electric contribution, *n* = 2 to the quadrupolar electric one. The contribution of all these modes to the total extinction cross-section can then be computed through their expansion coefficients:


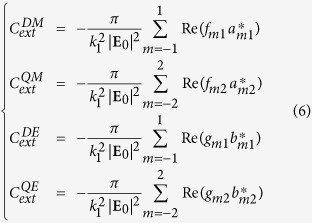


These expressions are used to plot the different mode extinctions in Fig. 2.

The complex valued extinction 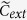 is defined^30^ as a function of the far electric field scattered by the structure 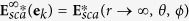 in the forward direction **e**_*k*_ of the incident plane wave given by the spherical angles (*θ*, *φ*), and is practically computed using the expansion coefficients:


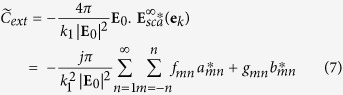


with 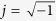.

As in the case of the extinction cross-section, the contribution of all magnetic and electric modes to the total complex extinction is computed by using the related expansion terms:


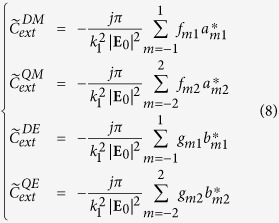


The complex valued extinction of the dipolar electric and the quadrupolar magnetic modes are used in the singular analysis to characterize their coupling.

### Fabrication of gold oligomers

The gold nanostructures are fabricated using electron beam lithography. Poly-methyl methacrylate (PMMA, 1%, 950 k in molecular weight) polymer is spin coated at 1000 rpm on the glass substrate to form a 60 nm in thickness layer and baked 5 min at 180 °C. An Aquasave conductive polymer is then spun at 2000 rpm and baked at 100 °C. This film dissipates the charges while exposing the sample to an electron beam (Vistec VB300, 100 kV, 1 nA, dose = 2500 μC/cm^2^ and up) to form the desired patterns. The substrate is then dipped in water for 30 s to dissolve the Aquasave coating. The PMMA film is developed using a cold process by soaking the substrate in an isopropanol:water (7:3) solution at 5 °C, for 100 s, and under ultrasonication. This cold development process is non-standard, extra high contrast and gives the higher resolution of the structures. Finally both chromium (5 nm) and gold (25 nm) are deposited (using a Semicore evaporator) on the substrate before performing a lift-off of the PMMA film using dichloromethane.

The same oligomer has been reproduced in a 5 μm in period regular lattice. This periodicity prevent radiative coupling between individual structures.

### Spectral measurement of incidence angle dependent extinction

**Figure 8:**
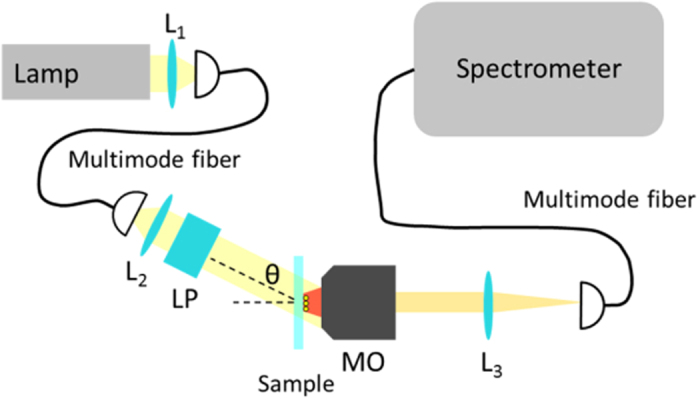
Schematics of the experimental set-up used to probe the 3 × 3 arrays of gold cylinders. The light emitted by a white lamp is coupled to a multimode fiber. The multimode fiber is out-coupled on a rotating board. To control the polarization of the incident white source, a linear polarizer (LP) is mounted on the rotating board. The beam illuminates the sample, and a microscope objective (MO) collimates the scattered and the incident light. The extinction signal is detected in a confocal manner, focusing the collimated beam onto another multimode fiber. The fiber is connected to a spectrometer, which analyzes the spectrum.

The spectral behavior of the samples shown in Fig. 6d has been measured with the set-up schematically displayed in Fig. 8. A white light source (Newport 67011) is coupled to a multimode fiber using a parabolic mirror, which acts like a lens (L_1_). The other end of the multimode fiber is fixed to a movable board. The light out-coupled from the fiber is collimated with a lens (L_2_). Then, it is linearly polarized using a linear polarizer (LP). Now, to know the angular position θ of the movable board, the latter is mounted on top of a goniometer. The alignment of the platform is done with respect to the normal direction of a sample holder, which is chosen to be the 0° of the goniometer. The sample holder is moved with a 3D nano-positioning system (PI, P-611.3), which is attached to three 1D translation stages. The substrate containing the 3 × 3 arrays is glued onto the sample holder. Then, the linearly polarized beam is shone onto the sample. The scattering of the arrays, as well as the direct illumination, are collected with a microscope objective (MO, Mitutoyo × 100, NA = 0.7, magnification of 100x). Since the sample is placed on the focal plane of the MO, the signal is collimated after MO. Then, a tube lens (L_3_) having a focal distance of 150 mm focuses the light down to a multimode fiber, which acts as a confocal filter. Finally, the fiber is then plugged into a spectrometer (Princeton Instr., IsoPlane SCT320), where the extinction spectra of the different measurements are retrieved. The confocal detection area is about 700 nm in diameter that allows measurements on single structures.

Then, in order to measure the extinction of a nanoarray, a double measurement is done. Keeping the same illumination always, two confocal measurements are done. Firstly, a background spectrum is recorded (I_b_). This background measurement takes into account the lamp spectrum, as well as the effect of all the other elements present in the set-up. Then, a measurement of the 3 × 3 structure in consideration is done (I). The extinction measurement is obtained using the following relation:


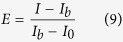


where I_0_ is a noise spectrum measurement, obtained without any illumination. All the extinction spectra measurements of these structures have been performed with an s-polarized incident plane wave at various incidence angles. Note that at each incidence angle, a new background measurement was carried out. The results depicted in Fig. 6c are obtained by averaging 5 extinction measurements of the same single structure.

## Additional Information

**How to cite this article**: Bakhti, S. *et al*. Fano-like resonance emerging from magnetic and electric plasmon mode coupling in small arrays of gold particles. *Sci. Rep.* **6**, 32061; doi: 10.1038/srep32061 (2016).

## Supplementary Material

Supplementary Information

## References

[b1] Kreibig U. & Vollmer M. Optical properties of metal clusters. (Springer, 1995).

[b2] Zhang J. & Zhang L. Nanostructures for surface plasmons. Adv. Opt. Photonics 4, 157–321 (2012).

[b3] Eustis S. & El-Sayed M. A. Why gold nanoparticles are more precious than pretty gold: Noble metal surface plasmon resonance and its enhancement of the radiative and nonradiative properties of nanocrystals of different shapes. Chem. Soc. Rev. 35, 209 (2006). 16505915 10.1039/b514191e

[b4] Bakhti S., Destouches N. & Tishchenko A. V. Analysis of plasmon resonances on a metal particle. J. Quant. Spectrosc. Radiat. Transf. 146, 113–122 (2014).

[b5] Mock J. J., Barbic M., Smith D. R., Schultz D. A. & Schultz S. Shape effects in plasmon resonance of individual colloidal silver nanoparticles. J. Chem. Phys. 116, 6755–6759 (2002).

[b6] Noguez C. Surface Plasmons on Metal Nanoparticles: The Influence of Shape and Physical Environment. J. Phys. Chem. C 111, 3806–3819 (2007).

[b7] Prodan E., Radloff C., Halas N. J. & Nordlander P. A Hybridization Model for the Plasmon Response of Complex Nanostructures. Science 302, 419–422 (2003). 14564001 10.1126/science.1089171

[b8] Nordlander P., Oubre C., Prodan E., Li K. & Stockman M. I. Plasmon Hybridization in Nanoparticle Dimers. Nano Lett. 4, 899–903 (2004).

[b9] Wang H., Brandl D. W., Nordlander P. & Halas N. J. Plasmonic Nanostructures: Artificial Molecules. Accounts Chem. Res. 40, 53–62 (2007). 10.1021/ar0401045 17226945

[b10] Anker J. N. . Biosensing with plasmonic nanosensors. Nat. Mater. 7, 442–453 (2008). 18497851 10.1038/nmat2162

[b11] Kneipp K., Moskovits M. & Kneipp H. Surface-Enhanced Raman Scattering: Physics and Applications. (Springer, 2006).

[b12] Boisselier E. & Astruc D. Gold nanoparticles in nanomedicine: preparations, imaging, diagnostics, therapies and toxicity. Chem. Soc. Rev. 38, 1759–1782 (2009). 19587967 10.1039/b806051g

[b13] Maier S. a. . Plasmonics—A Route to Nanoscale Optical Devices (Advanced Materials, 2001, 13, 1501). Adv. Mater. 15, 562–562 (2003).

[b14] Liu N. . Magnetic Plasmon Formation and Propagation in Artificial Aromatic Molecules. Nano Lett. 12, 364–369 (2012). 22122612 10.1021/nl203641z

[b15] Alù A. & Engheta N. Dynamical theory of artificial optical magnetism produced by rings of plasmonic nanoparticles. Phys. Rev. B 78, 085112 (2008).

[b16] Alù A., Salandrino A. & Engheta N. Negative effective permeability and left-handed materials at optical frequencies. Opt. Express 14, 1557–1567 (2006). 19503482 10.1364/oe.14.001557

[b17] Monticone F. & Alù A. The quest for optical magnetism: from split-ring resonators to plasmonic nanoparticles and nanoclusters. J. Mater. Chem. C 2, 9059–9072 (2014).

[b18] Zambrana-Puyalto X., Vidal X., Juan M. L. & Molina-Terriza G. Dual and anti-dual modes in dielectric spheres. Opt. Express 21, 17520 (2013). 23938623 10.1364/OE.21.017520

[b19] Zambrana-Puyalto X. & Bonod N. Tailoring the chirality of light emission with spherical Si-based antennas. Nanoscale 8, 10441–10452 (2016). 27141982 10.1039/c6nr00676k

[b20] Smith D. R., Pendry J. B. & Wiltshire M. C. K. Metamaterials and Negative Refractive Index. Science 305, 788–792 (2004). 15297655 10.1126/science.1096796

[b21] Grigorenko A. N. . Nanofabricated media with negative permeability at visible frequencies. Nature 438, 335–338 (2005). 16292306 10.1038/nature04242

[b22] Veselago V., Braginsky L., Shklover V. & Hafner C. Negative Refractive Index Materials. J. Comput. Theor. Nanosci. 3, 189–218 (2006).

[b23] Luk’yanchuk B. . The Fano resonance in plasmonic nanostructures and metamaterials. Nat. Mater. 9, 707–715 (2010). 20733610 10.1038/nmat2810

[b24] Garrido Alzar C. L., Martinez M. A. G. & Nussenzveig P. Classical analog of electromagnetically induced transparency. Am. J. Phys. 70, 37 (2002).

[b25] Joe Y. S., Satanin A. M. & Kim C. S. Classical analogy of Fano resonances. Phys. Scr. 74, 259 (2006).

[b26] Lassiter J. B. . Designing and Deconstructing the Fano Lineshape in Plasmonic Nanoclusters. Nano Lett. 12, 1058–1062 (2012). 22208801 10.1021/nl204303d

[b27] Fan S., Suh W. & Joannopoulos J. D. Temporal coupled-mode theory for the Fano resonance in optical resonators. J. Opt. Soc. Am. 20, 569 (2003). 10.1364/josaa.20.000569 12630843

[b28] Ruan Z. & Fan S. Temporal Coupled-Mode Theory for Fano Resonance in Light Scattering by a Single Obstacle^†^. J. Phys. Chem. C 114, 7324–7329 (2010).

[b29] Hsu C. W., DeLacy B. G., Johnson S. G., Joannopoulos J. D. & Soljačić M. Theoretical Criteria for Scattering Dark States in Nanostructured Particles. Nano Lett. 14, 2783–2788 (2014). 24805881 10.1021/nl500340n

[b30] Bakhti S., Destouches N. & Tishchenko A. V. Coupled Mode Modeling To Interpret Hybrid Modes and Fano Resonances in Plasmonic Systems. ACS Photonics 2, 246–255 (2015).

[b31] Forestiere C., Dal Negro L. & Miano G. Theory of coupled plasmon modes and Fano-like resonances in subwavelength metal structures. Phys. Rev. B 88, 155411 (2013).

[b32] Hopkins B., Poddubny A. N., Miroshnichenko A. E. & Kivshar Y. S. Revisiting the physics of Fano resonances for nanoparticle oligomers. Phys. Rev. 88, 053819 (2013).

[b33] Grigoriev V. . Singular analysis of Fano resonances in plasmonic nanostructures. Phys. Rev. 88, 063805 (2013).

[b34] Gómez D. E. . The Dark Side of Plasmonics. Nano Lett. 13, 3722–3728 (2013). 23802620 10.1021/nl401656e

[b35] Lovera A., Gallinet B., Nordlander P. & Martin O. J. F. Mechanisms of Fano Resonances in Coupled Plasmonic Systems. ACS Nano 7, 4527–4536 (2013). 23614396 10.1021/nn401175j

[b36] Frimmer M., Coenen T. & Koenderink A. F. Signature of a Fano Resonance in a Plasmonic Metamolecule’s Local Density of Optical States. Phys. Rev. Lett. 108, 077404 (2012). 22401256 10.1103/PhysRevLett.108.077404

[b37] McLeod A. . Nonperturbative Visualization of Nanoscale Plasmonic Field Distributions via Photon Localization Microscopy. Phys. Rev. Lett. 106, 037402 (2011). 21405296 10.1103/PhysRevLett.106.037402

[b38] Zhang Z. . Manipulating Nanoscale Light Fields with the Asymmetric Bowtie Nano-Colorsorter. Nano Lett. 9, 4505–4509 (2009). 19899744 10.1021/nl902850f

[b39] Sheikholeslami S. N., García-Etxarri A. & Dionne J. A. Controlling the Interplay of Electric and Magnetic Modes via Fano-like Plasmon Resonances. Nano Lett. 11, 3927–3934 (2011). 21819059 10.1021/nl202143j

[b40] Shafiei F. . A subwavelength plasmonic metamolecule exhibiting magnetic-based optical Fano resonance. Nat. Nanotechnol. 8, 95–99 (2013). 23353675 10.1038/nnano.2012.249

[b41] Mackowski D. W. Analysis of Radiative Scattering for Multiple Sphere Configurations. Proc. R. Soc. Lond. Ser. Math. Phys. Sci. 433, 599–614 (1991).

[b42] Xu Y. Electromagnetic scattering by an aggregate of spheres. Appl. Opt. 34, 4573 (1995). 21052290 10.1364/AO.34.004573

[b43] Haus H. A. & Huang W. Coupled-mode theory. Proc. IEEE 79, 1505–1518 (1991).

[b44] Bakhti S., Destouches N. & Tishchenko A. V. In Reviews in Plasmonics 2015 (ed. Geddes C. D.) 19–49 (Springer International Publishing, 2016).

[b45] Bakhti S., Destouches N. & Tishchenko A. V. Singular Representation of Plasmon Resonance Modes to Optimize the Near- and Far-Field Properties of Metal Nanoparticles. Plasmonics 10, 1391–1399 (2015).

[b46] De Waele R., Koenderink A. F. & Polman A. Tunable Nanoscale Localization of Energy on Plasmon Particle Arrays. Nano Lett. 7, 2004–2008 (2007).

[b47] Devilez A., Stout B. & Bonod N. Mode-balancing far-field control of light localization in nanoantennas. Phys. Rev. B 81, 245128 (2010).

[b48] Koenderink A. F., Hernández J. V., Robicheaux F., Noordam L. D. & Polman A. Programmable Nanolithography with Plasmon Nanoparticle Arrays. Nano Lett. 7, 745–749 (2007). 17315939 10.1021/nl0630034

[b49] Mirin N. A., Bao K. & Nordlander P. Fano Resonances in Plasmonic Nanoparticle Aggregates^†^. J. Phys. Chem. A 113, 4028–4034 (2009). 19371111 10.1021/jp810411q

[b50] Fan J. A. . Self-Assembled Plasmonic Nanoparticle Clusters. Science 328, 1135–1138 (2010). 20508125 10.1126/science.1187949

[b51] Mackowski D. In The Mie Theory (eds Hergert W. & Wriedt T.) 223–256 (Springer Berlin Heidelberg, 2012).

[b52] Johnson P. B. & Christy R. W. Optical Constants of the Noble Metals. Phys. Rev. B 6, 4370–4379 (1972).

[b53] Doicu A., Wriedt T. & Eremin Y. A. Light Scattering by Systems of Particles-Null-Field Method with Discrete Sources: Theory and (Springer, 2006).

[b54] Mackowski D. W. Calculation of total cross sections of multiple-sphere clusters. J. Opt. Soc. Am. 11, 2851–2861 (1994).

