# Supplementary material for: Fano-like resonance emerging from magnetic and electric plasmon mode coupling in small arrays of gold particles
Source: Sci Rep. 2016 Sep 1;6:32061. doi: 10.1038/srep32061 (PMC5007502; doi:10.1038/srep32061)
Supplement: Supplementary Information [file srep32061-s1.pdf]

Supplementary Information

Manuscript: Fano-like resonance emerging from magnetic and electric plasmon mode coupling in small arrays of gold particles

Authors: S. Bakhti, A.V. Tishchenko, X. Zambrana-Puyalto, N. Bonod, S.D. Dhuey, P.J. Schuck, S. Cabrini, S. Alayoglu, N. Destouches

1. Scattering coefficients

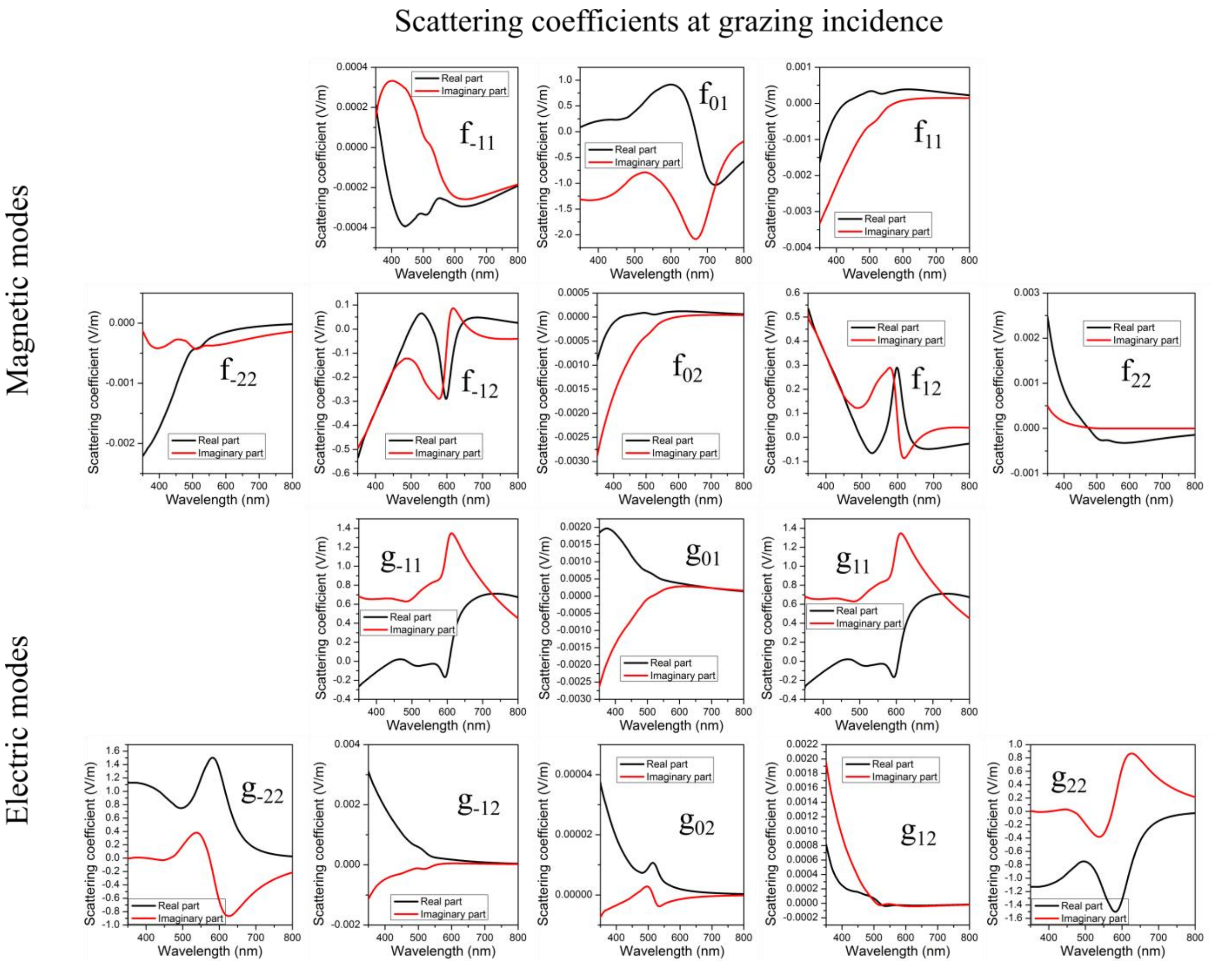

Figure S1. Plot of the dipolar and quadrupolar scattering coefficients of the array of 3x3 gold spheres illuminated at grazing incidence.

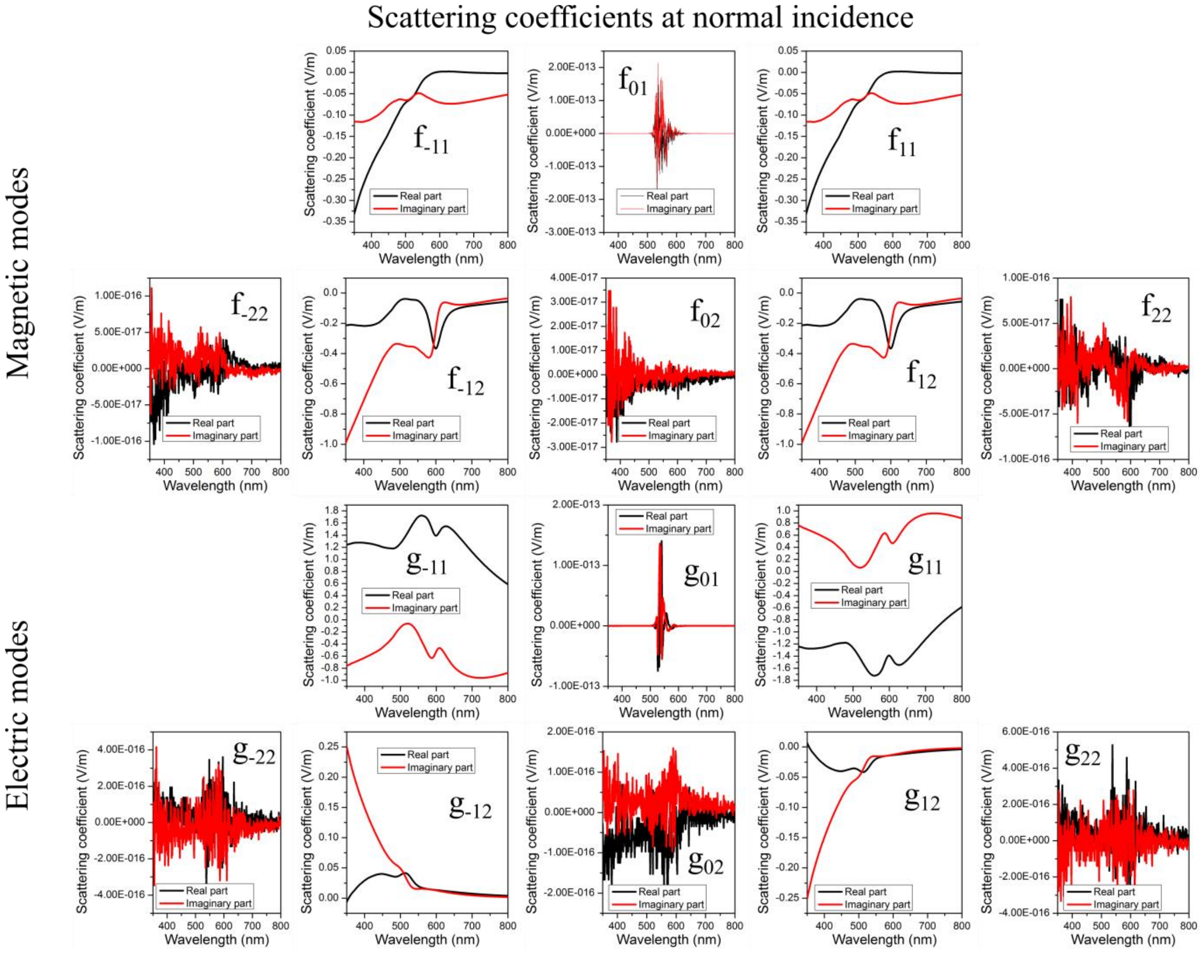

Figure S2. Plot of the dipolar and quadrupolar scattering coefficients of the array of 3x3 gold spheres illuminated at normal incidence.

2. Phase of complex valued extinction

The complex valued extinction of a given resonance mode is analytically described as a singular function of the angular frequency:

$$\tilde{C}_{ext}(\omega) = \frac{a_p}{\omega - \omega_p} \tag{S1}$$

where a_p and ω_p are the complex valued resonance amplitude and eigen angular frequency, respectively. The resonance line profile directly derives from particular phase relations between the resonance mode and the driving excitation. At resonance position, the resonance phase φ_r is related to the amplitude phase $\varphi_a = \varphi_r + \pi/2$. As an example, the real and imaginary parts of the complex extinction spectrum are plotted in Figure S3 for various phases at resonance φ_r, the resonance position being fixed to 600 nm and its HWHM to 30 nm. The imaginary part of the complex extinction, corresponding to the extinction cross-section, has a Lorentzian line shape when $\varphi_r = \pi/2$ that corresponds to a classical resonator behavior.

Paying attention to the dipolar electric mode (Fig. 3a), the characteristic dip in its extinction spectrum appears to result from destructive interferences between the two hybrid modes. Indeed the mode -, with an absolute phase of -86° at resonance (being out of phase with a usual resonance), has a Lorentzian shape with a negative contribution to the total extinction. The superposition of this negative contribution with the broader mode +, having a phase of 97° at resonance and a nearly Lorentzian profile, leads to the dip in the total mode extinction. Regarding the quadrupolar magnetic mode (Fig. 3b), the high asymmetry in its extinction spectrum is essentially imposed by the mode – contribution having a 11° absolute phase at resonance, which is nearly $-\pi/2$ phase shifted compared to a usual Lorentzian profile. The presence of these two contributions resulting from hybrid modes + and – respectively to the electric and magnetic modes is a direct effect of the coupling between these modes.

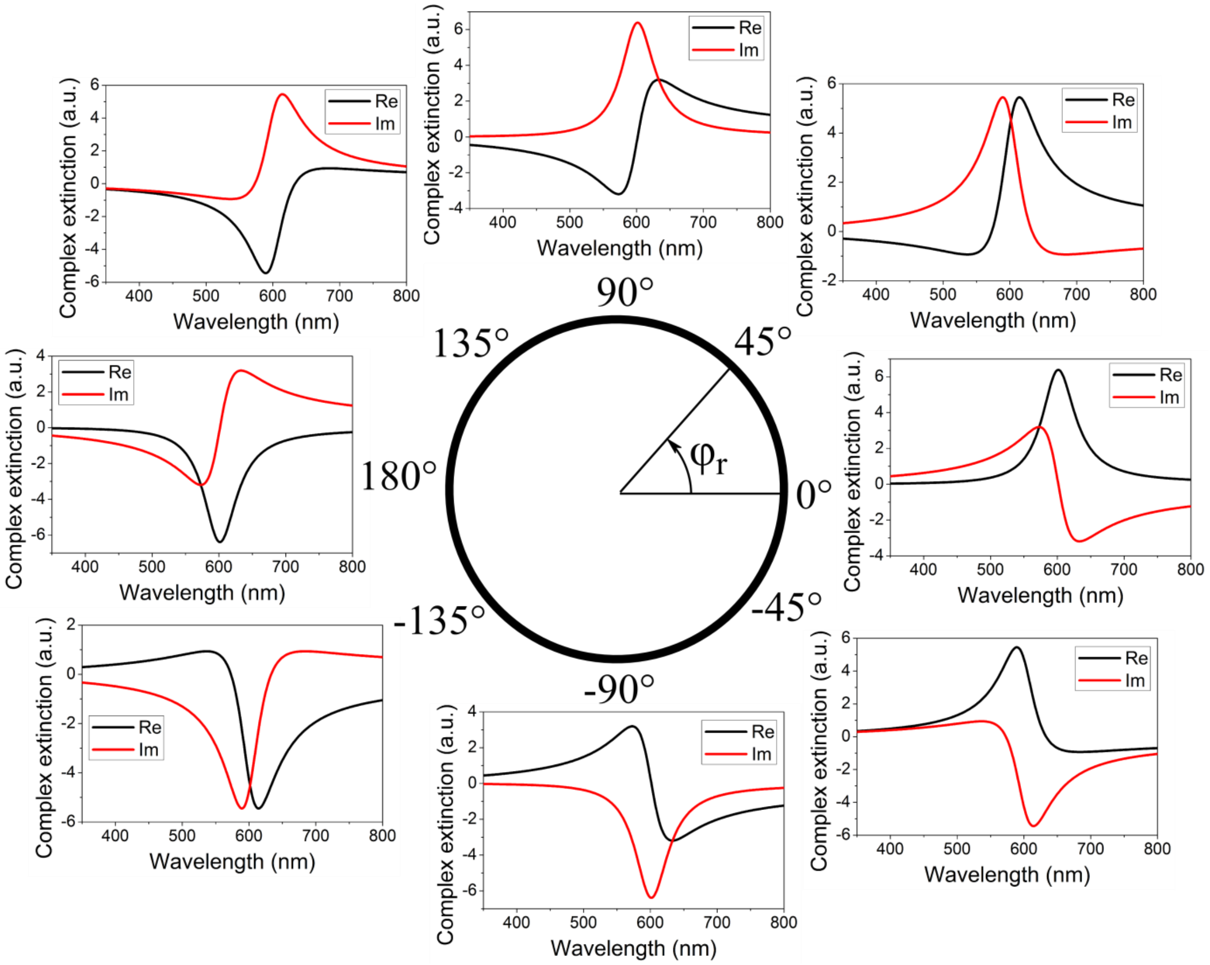

Figure S3. Plot of the real and imaginary parts of the complex extinction spectrum for various phases at resonance φ_r . The resonance position is fixed to 600 nm and its HWHM to 30 nm.

3. Far-field patterns

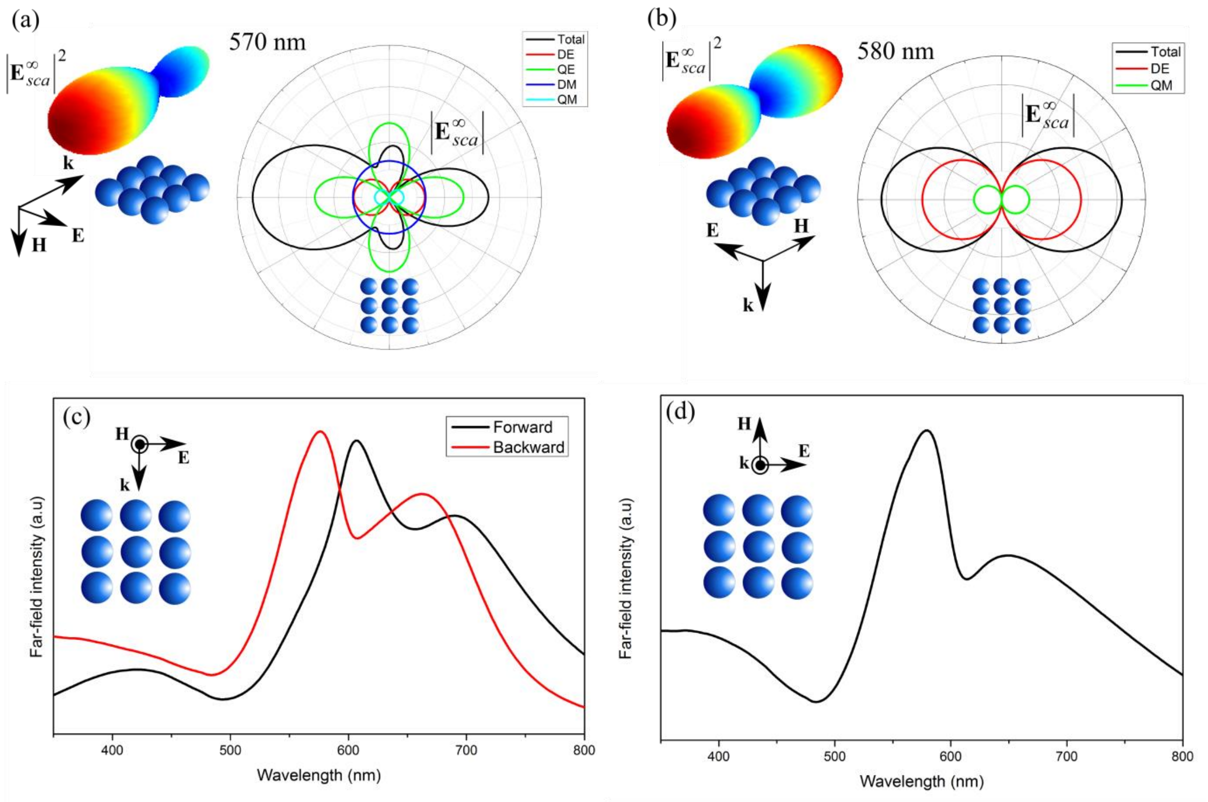

Figure S4. Far-field scattered by the structure under (a) grazing and (b) normal incidence, with the contribution of the different modes to the total scattering. (c) Wavelength dependence on the far-field intensity in forward and backward directions under grazing incidence. (d) Wavelength dependence on the maximum far-field intensity under normal incidence.

4. FDTD near-field simulations

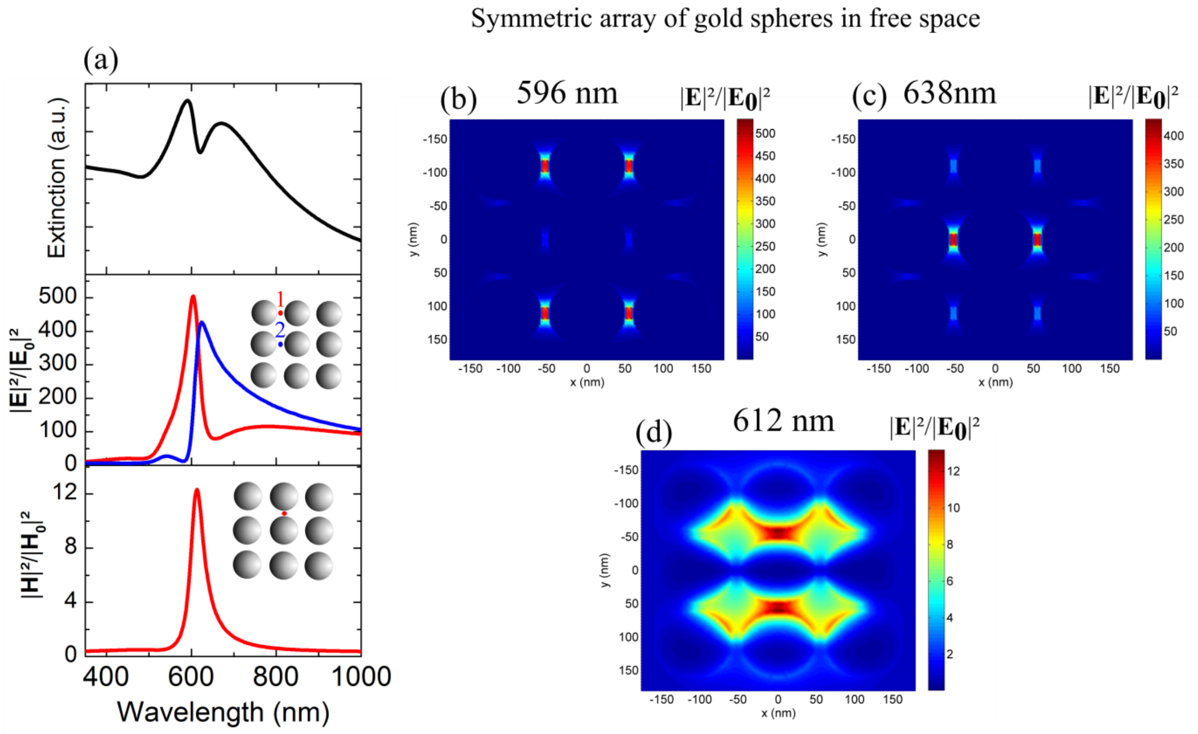

Figure S5. FDTD simulations of a regular array of gold spheres in vacuum. The particle radius is 50 nm and the gap separating them is 10 nm. (a) Extinction spectrum of the structure with the electric and magnetic field enhancement at different locations. Mapping of the electric field intensity at (b) 596 and (c) 638 nm, and (d) the magnetic field at 612 nm.

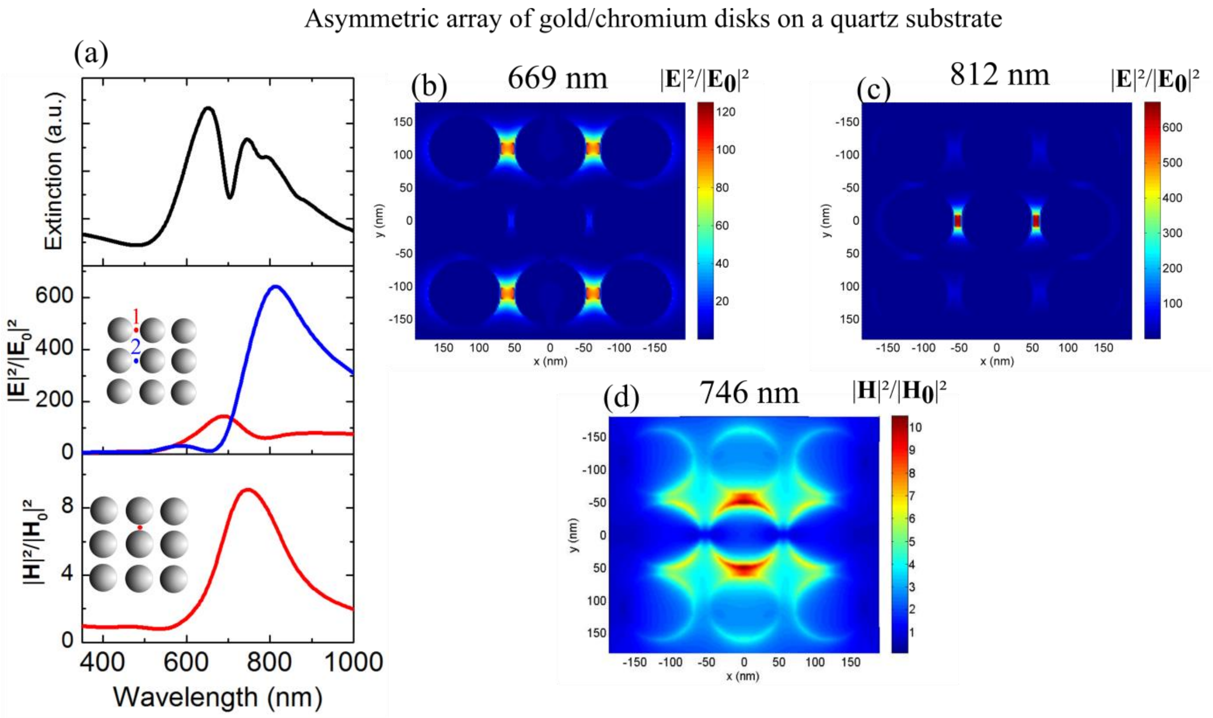

Figure S6. FDTD simulations of an asymmetric array of gold/chromium disks on a quartz substrate. This configuration corresponds to the experimental. (a) Extinction spectrum of the structure with the electric and magnetic field enhancement at different locations. Mapping of the electric field intensity at (b) 669 and (c) 812 nm, and (d) the magnetic field at 746 nm.

5. Effect of the structure geometry and environment

If the experimental structure qualitatively behaves like the first one considered in the theoretical study, differences in their spectral line shape exist and result from changes in the geometrical configurations. FDTD simulations with gradually changing oligomers (Figure 6e) show how geometrical features affect the structure's optical extinction. Breaking the periodicity of the regular array of gold spheres leads to a strengthening of the Fano dip in the extinction spectrum. We can note in this case a good agreement between analytical and numerical calculations regarding both the optical cross-section and the near-field enhancement (shown in Fig S6 in Supplementary information). Replacing spheres by disks not only enhances the strengthening of the Fano dip but also induces a significant redshift of resonance. Using fully gold nano-disks clearly increases the amplitude of optical resonances, but adding a lossy bounding chromium layer clearly causes an important attenuation of the Fano dip as well as a slight broadening of the main resonance band. The substrate do not induces noticeable shift in the Fano dip position but rather its strengthening and a change in the relative amplitude of the extinction peaks from either side of the dip. The weak effect of the substrate on the resonance position may be attributed to the bi-metallic nature of the particles. Then gold particles are not directly deposited on the quartz substrate but on chromium disks, acting as a spacer preventing a significant substrate induced hybridization of the plasmon modes.[1,2] Finally, the random inhomogeneity and roughness of the fabricated particles may also affect in different ways the coupling processes in the structure compared to a perfect geometry. Differences between experimental measurements (Fig. 6a) and numerical simulations (Fig. 6b), and especially the zero extinction obtained experimentally but not numerically at 700 nm at normal incidence, may result from such inhomogeneity.

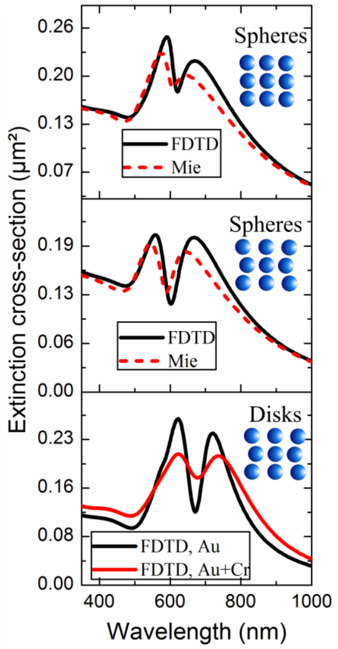

Figure S7. Comparisons between analytical and numerical calculations in the cases of periodic and non-periodic oligomers of gold nano-spheres in air, and numerical FDTD simulations of the experimental oligomer in free space with and without the chromium disks.

6. Gold heptamer

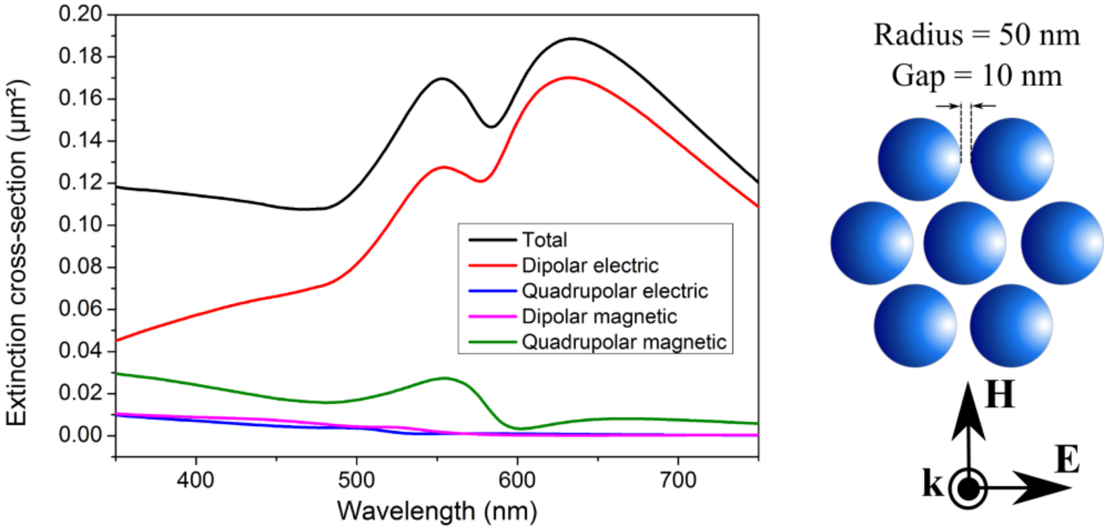

Figure S8. Contribution of different magnetic and electric modes to the total extinction of a gold heptamer at normal incidence.

7. Gold dielectric permittivity

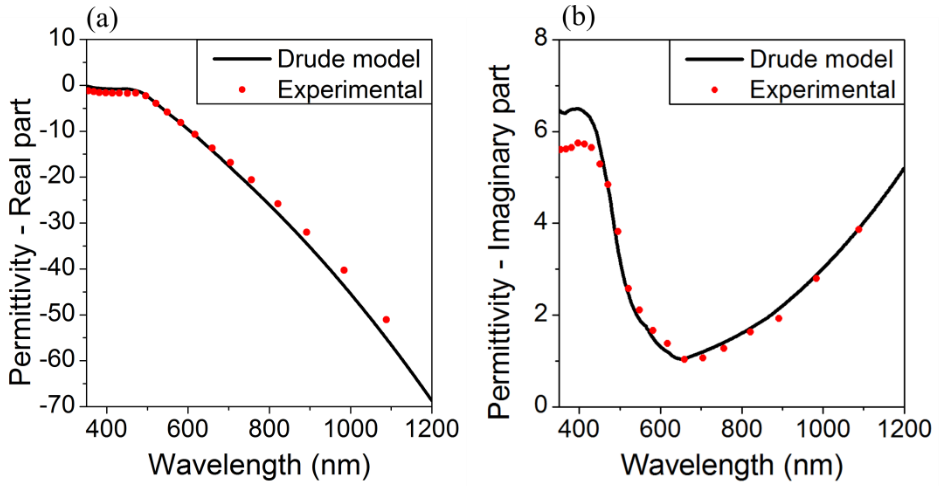

Figure S9. (a) Real and (b) imaginary part of the gold dielectric permittivity, as obtained using the semi-analytical Drude model and from experimental data (Ref. [3]).

References

1. Knight, M. W., Wu, Y., Lassiter, J. B., Nordlander, P. & Halas, N. J. Substrates Matter: Influence of an Adjacent Dielectric on an Individual Plasmonic Nanoparticle. *Nano Lett.* **9,** 2188–2192 (2009).
2. Lermé, J. *et al.* Optical Properties of a Particle above a Dielectric Interface: Cross Sections, Benchmark Calculations, and Analysis of the Intrinsic Substrate Effects. *J. Phys. Chem. C* **117,** 6383–6398 (2013).
3. Johnson, P. B. & Christy, R. W. Optical Constants of the Noble Metals. *Phys. Rev. B* **6,** 4370–4379 (1972).
